# Novel adenovirus detected in captive bottlenose dolphins (*Tursiops truncatus*) suffering from self-limiting gastroenteritis

**DOI:** 10.1186/s12917-015-0367-z

**Published:** 2015-03-07

**Authors:** Consuelo Rubio-Guerri, Daniel García-Párraga, Elvira Nieto-Pelegrín, Mar Melero, Teresa Álvaro, Mónica Valls, Jose Luis Crespo, Jose Manuel Sánchez-Vizcaíno

**Affiliations:** VISAVET Center and Animal Health Department, Veterinary School, Complutense University of Madrid, Av Puerta del Hierro s/n, 28040 Madrid, Spain; Veterinary Services, Oceanographic Aquarium of the Ciudad de las Artes y las Ciencias, C/ Junta de Murs i Valls s/n, 46023 Valencia, Spain

**Keywords:** Adenovirus, Cetacean, Bottlenose dolphin, *Tursiops truncatus*

## Abstract

**Background:**

Adenoviruses are common pathogens in vertebrates, including humans. In marine mammals, adenovirus has been associated with fatal hepatitis in sea lions. However, only in rare cases have adenoviruses been detected in cetaceans, where no clear correlation was found between presence of the virus and disease status.

**Case presentation:**

A novel adenovirus was identified in four captive bottlenose dolphins with self-limiting gastroenteritis. Viral detection and identification were achieved by: PCR-amplification from fecal samples; sequencing of partial adenovirus *polymerase (pol)* and *hexon* genes; producing the virus in HeLa cells, with PCR and immunofluorescence detection, and with sequencing of the amplified *pol* and *hexon* gene fragments. A causative role of this adenovirus for gastroenteritis was suggested by: 1) we failed to identify other potential etiological agents; 2) the exclusive detection of this novel adenovirus and of seropositivity for canine adenoviruses 1 and 2 in the four sick dolphins, but not in 10 healthy individuals of the same captive population; and 3) the virus disappeared from feces after clinical signs receded. The partial sequences of the amplified fragments of the *pol* and *hexon* genes were closest to those of adenoviruses identified in sea lions with fatal adenoviral hepatitis, and to a Genbank-deposited sequence obtained from a harbour porpoise.

**Conclusion:**

These data suggest that adenovirus can cause self-limiting gastroenteritis in dolphins. This adenoviral infection can be detected by serology and by PCR detection in fecal material. Lack of signs of hepatitis in sick dolphins may reflect restricted tissue tropism or virulence of this adenovirus compared to those of the adenovirus identified in sea lions. Gene sequence-based phylogenetic analysis supports a common origin of adenoviruses that affect sea mammals. Our findings suggest the need for vigilance against adenoviruses in captive and wild dolphin populations.

## Background

Adenoviruses are common pathogens of vertebrates [1] that were first discovered in human adenoids [2], and were soon identified as a cause of canine hepatitis [3]. These icosahedral non-enveloped, double-stranded DNA viruses have genomes that range from 26 to 45 kbp [4], and have been demonstrated in all vertebrate classes [1,5]. Most adenoviral species show quite restricted host specificity and tend to be associated with a typical pathology [5]; for example, human adenovirus (HAdV) C causes respiratory disease and HAdV-D provokes conjunctivitis, whereas these two pathologies can also be the result of HAdV-B infection. In contrast, HAdV-F and HAdV-G produce gastroenteritis in most cases [1]. Similarly to human adenoviruses, other adenoviruses that affect mammals (forming the *Mastadenovirus* genus) have been reported to cause respiratory, ocular and gastrointestinal pathologies, although some present as hepatitis [3] or encephalitis as the chief manifestations [5].

In addition to their role in pathology, adenoviruses are very important vectors in the gene therapy of genetic disorders and cancer [6], as they can accommodate a large DNA cargo, exhibit tropisms for multiple organs and can be engineered to decrease virulence. Nonetheless, they still present toxicity problems [7], which has led to investigation of the potential of using animal adenoviruses as vectors for gene delivery to humans [8-10]. In line with this, the identification of new animal adenoviruses, in addition to being interesting from an animal health perspective may be promising for gene therapy.

Sea lions are the only marine mammals in which adenoviruses have been recognized as pathogens. Adenovirus-like viral particles have been long since associated with hepatitis in stranded California sea lions (*Zalophus californianus)* [11,12]. More recently, a novel adenovirus (otarine adenovirus 1) was isolated from two stranded California sea lions with fatal hepatitis [13]. This adenovirus caused an outbreak of fatal hepatitis and enteritis in three captive sea lions of different species: California sea lion (*Zalophus californianus)*, South African fur seal (*Arctocephalus pusillus)* and South American sea lion (*Otaria flavescens)* [14]. In rare cases, adenoviruses have been isolated from gastrointestinal samples of other marine mammals, including a sei whale (*Balaenoptera borealis*) [15], two bowhead whales (*Balaena mysticetus*) [16] and a beluga whale (*Delphinapterus leucas*) [17]. Serological studies in Canadian fauna have also revealed antibodies against canine adenovirus 2 in 17% of the walruses (*Odobenus rosmarus*) examined [18]. However, only in the case of sea lion hepatitis, has a clear association been established between the presence of virus and disease status. The partial sequence of the adenoviral DNA *polymerase* (*pol)* gene deposited in the Genbank (JN377908) was annotated as having been obtained from a harbour porpoise (*Phocoena phocoena*). However, there is no further information or referred publication available.

Here we identify a novel adenovirus in fecal samples of four captive bottlenose dolphins (*Tursiops truncatus*) which presented with self-limiting gastroenteritis. Gastric lesions, ulceration and parasitism are common in captive and free-ranging dolphins [19-21]. However, reports of dolphin gastroenteritis are rare and the disease has never been associated with adenovirus. Pathological evidence for gastroenteritis has been reported [22] in two necropsies of common dolphins from the Black Sea *(Delphinus delphis ponticus)* that showed evidence of systemic infection with *Cetacean morbillivirus* infection. Nevertheless, infections with this virus do not typically manifest as gastroenteritis and instead affect primarily the lungs and brain [23]. Fatal gastroenteritis and toxic shock-like syndrome in dolphins has been attributed to enterotoxigenic *Staphylococcus aureus* [24]. This animal concomitantly suffered brucellar osteomyelitis and was treated with antibiotics for nearly 1 year.

The present report describes several lines of evidence suggesting that adenovirus can be responsible for gastroenteritis in dolphins. Sequencing of PCR-amplified regions of adenoviral DNA (*pol)* and *hexon* genes revealed genetic closeness, but was not identical with the previously deposited sequences of sea lions and harbour porpoise adenoviruses. This suggests a close common ancestral origin of these viruses in marine mammals.

## Case presentation

**Figure 1:**
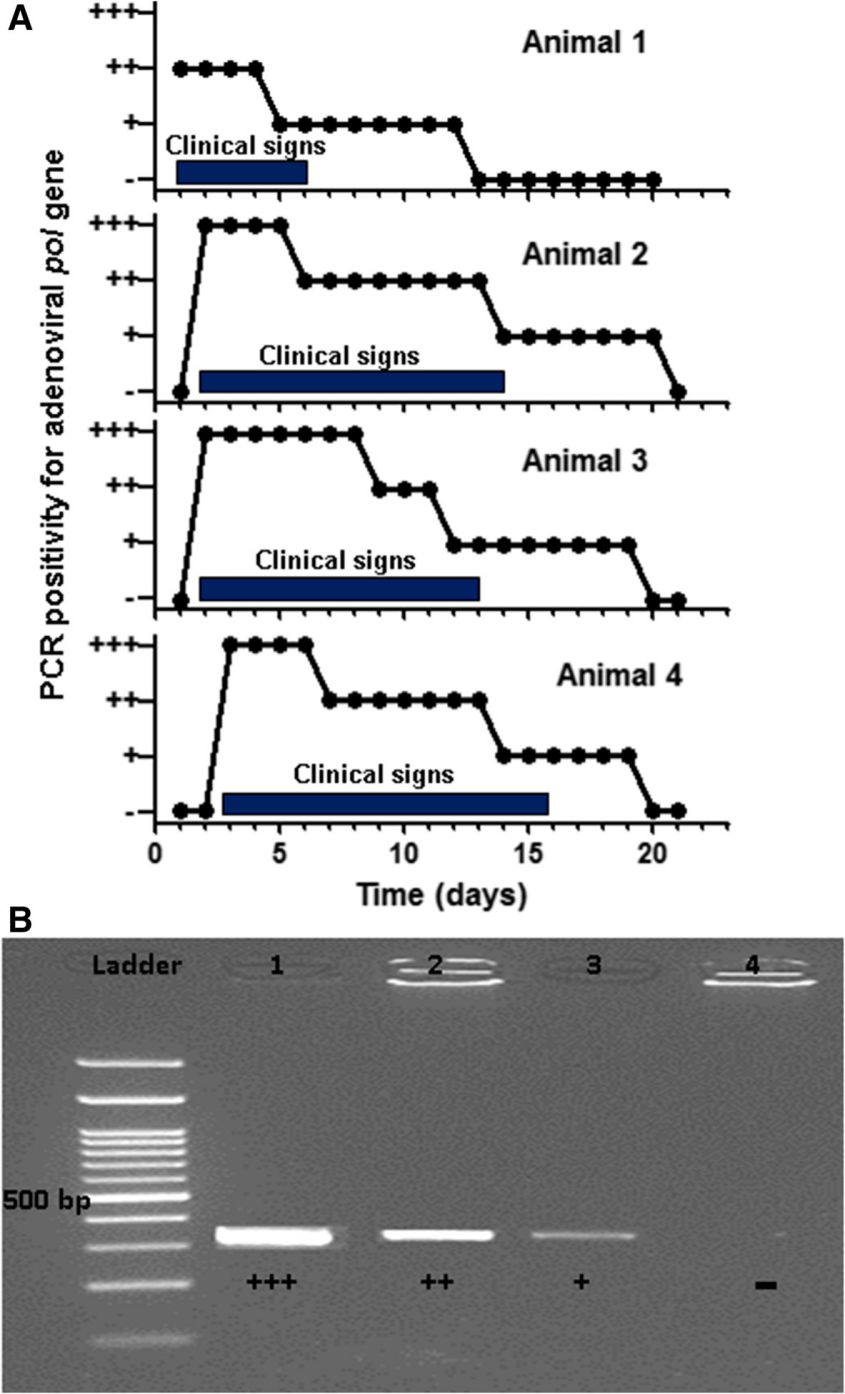
**Presence of adenoviral DNA in fecal samples of four diseased bottlenose dolphins. A)** Variation in the intensity of the adenoviral *polymerase (pol)* amplicon across different diseased animals and samples taken at the indicated times from the same animal. Black bars indicate the period during which each animal exhibited clinical manifestations. Day 1 was the day on which the first animal became overtly sick with vomiting and diarrhea. **B)** Representative results showing four levels of band intensity (- / + / ++ / +++) for the PCR amplicons of a region of the adenoviral *pol* gene. Line 1 corresponds to an amplification prepared from a fecal sample taken on day 2 from animal 2; Line 2, from a fecal sample taken on day 1 from animal 1; Line 3, on day 15 from animal 3; and Line 4, on day 20 from animal 4, used as negative controls. On the DNA ladder, the band of 500 bp is indicated.

At the end of September 2013, four captive bottlenose dolphins (*Tursiops truncatus*) in a total population of 14 individuals, all born at the Oceanográfic water park (www.cac.es/oceanografic) in the City of the Arts and the Sciences, Valencia (Spain), presented with anorexia, diarrhoea and vomiting. The four animals, aged 4-10 years, displayed no cough, respiratory disturbances or conjunctival infection. The other 10 dolphins in the same cohort remained healthy throughout this study. As soon as clinical signs became evident in the sick animals, they were isolated in a separate pool. The clinical signs of one dolphin (animal 1) appeared to be the mildest and it recovered in 1 week without treatment; in Figure 1A, the dark horizontal bar marks the period during which clinical manifestations were present. The other three dolphins (animals 2-4) were more severely affected and were administered oral rehydration therapy to compensate for fluid lost through vomiting and diarrhoea. They underwent longer disease-manifesting periods (Figure 1A). Day 1 was the day on which the first animal became overtly sick with vomiting and diarrhea.

Blood and fecal samples, obtained by rectal cannulation, were subjected to diagnostic tests. Animal sampling were conducted according to Spanish and European regulations (RD 53/2013, Directive 2010/63/UE) on animal welfare. No abnormalities were observed in the complete blood count, serum biochemistry or activity of serum enzymes such us aspartate and alanine aminotransferases. Blood samples gave negative serological results for several viruses (parvovirus, parainfluenza, coronavirus, influenza A) and for *Leptospira interrogans* (tests carried out by Penta Laboratories, Alicante, Spain). The microbiological analysis of feces performed at the Central Laboratory for Animal Health (Algete, Madrid, Spain) failed to reveal any bacterial pathogens using standard microbial growth assays in either sick or healthy animals. Fecal material also yielded negative results in a test for rotavirus antigen (Penta Laboratories) and in the fast immunoassays to detect canine parvovirus (VetScan Canine Parvovirus Rapid Test kit, Abaxis, Union City, CA, USA), bovine rotavirus, coronavirus and cryptosporidium (FASTest® D4T bovine; MEGACOR Diagnostik, Hörbranz, Austria), and in a real-time PCR (qPCR) assay for calicivirus [25]. The only differential finding between diseased and healthy animals was seropositivity for antibodies against canine adenoviruses 1 and 2 (ELISA kit D1003-AB01, European Veterinary Laboratory, The Netherlands) [26]. These antibodies were found exclusively in all four sick animals during the disease manifestation period (tested on days 2-4 of this period in animals 1-3, and on day 10 in animal 4). The other 10 healthy animals did not give a positive test (assayed on days 3-20 from the beginning of the outbreak; one animal was tested on both days 3 and 20). Before the outbreak, all 14 animals were seronegative for canine adenoviruses 1 and 2 (tested retrospectively on serum collected and kept frozen <4 months, usually around 2 months). Based on retrospective sampling, one sick animal was negative 15 days before disease, and a healthy animal was negative 5 days before the beginning of the outbreak). These findings led us to search for adenoviruses in the fecal samples collected from all 14 animals in the cohort from the beginning of the outbreak. The feces of healthy animals were collected daily the first 5 days of the outbreak and then every 5-10 days during the outbreak. Fecal sampling in the diseased animals was carried out daily for 21 days from the beginning of the outbreak and then every 5 days for another 10 days. Afterward fecal samples were collected from all animals every 15 days for 3 months.

To detect adenoviral DNA in feces, parts of the adenoviral *DNA polymerase* (*pol*) and *hexon* genes were PCR-amplified using degenerate consensus primers. For *pol* amplification, a previously described [27] nested PCR assay was performed exactly as reported, on 25 μl the of amplification reaction. For the first PCR reaction 1 μg of DNA extracted from fecal samples with High Pure PCR Template Preparation Kit (Roche Diagnostics GmbH, Mannheim, Germany) was used as a template. The same volumes of PCR reaction and amount of DNA were used for the amplification of the *hexon* sequence, and previously reported PCR assay was followed exactly [28].

The results of these PCR assays were analyzed by agarose gel electrophoresis and staining with SYBR Safe stain (Invitrogen, Carlsbad, CA, USA). All sick animals were positive for the expected *pol* amplicon (320 bp) (Figure 1B) and the *hexon* amplicon (435 bp, not shown), while all healthy animals were negative for both. The identity of the amplified products was confirmed by purification (QIAquick PCR Purification kit, Qiagen, Hilden, Germany) and identified by Sanger sequencing (ABI Prism 3730, from Applied Biosystems, Foster City, CA, USA) using one of the consensus primers utilized in the amplification step as the sequencing primer.

Comparison of the amplification reactions on the same amounts of DNA from the fecal samples collected from different animals and from the same animal on distinct days revealed variable band intensities. These intensities could be visually graded semi-quantitatively as negative (-), low (+), intermediate (++) or high (+++) (shown for *pol* in Figure 1B). Figure 1A plots these intensities for the *pol* band in relation to the presence of disease manifestations in each animal. Band intensity was strongest as soon as clinical signs were patent, and they remained at this initial high level for 4-7 days, finally decreasing before clinical signs subsided. The signal decreased to the lowest level around the same time as the clinical signs disappeared. Nevertheless, the virus remained detectable at low levels in feces for approximately 1 week after animals no longer manifested clinical signs. This suggests that, in order to prevent contagion, isolation of diseased animals should continue at least for 1 week after clinical cure. It is noteworthy that the animal with the mildest disease manifestations (animal 1) exhibited lower band intensity at peak infection than the more severely affected animals.

**Figure 2:**
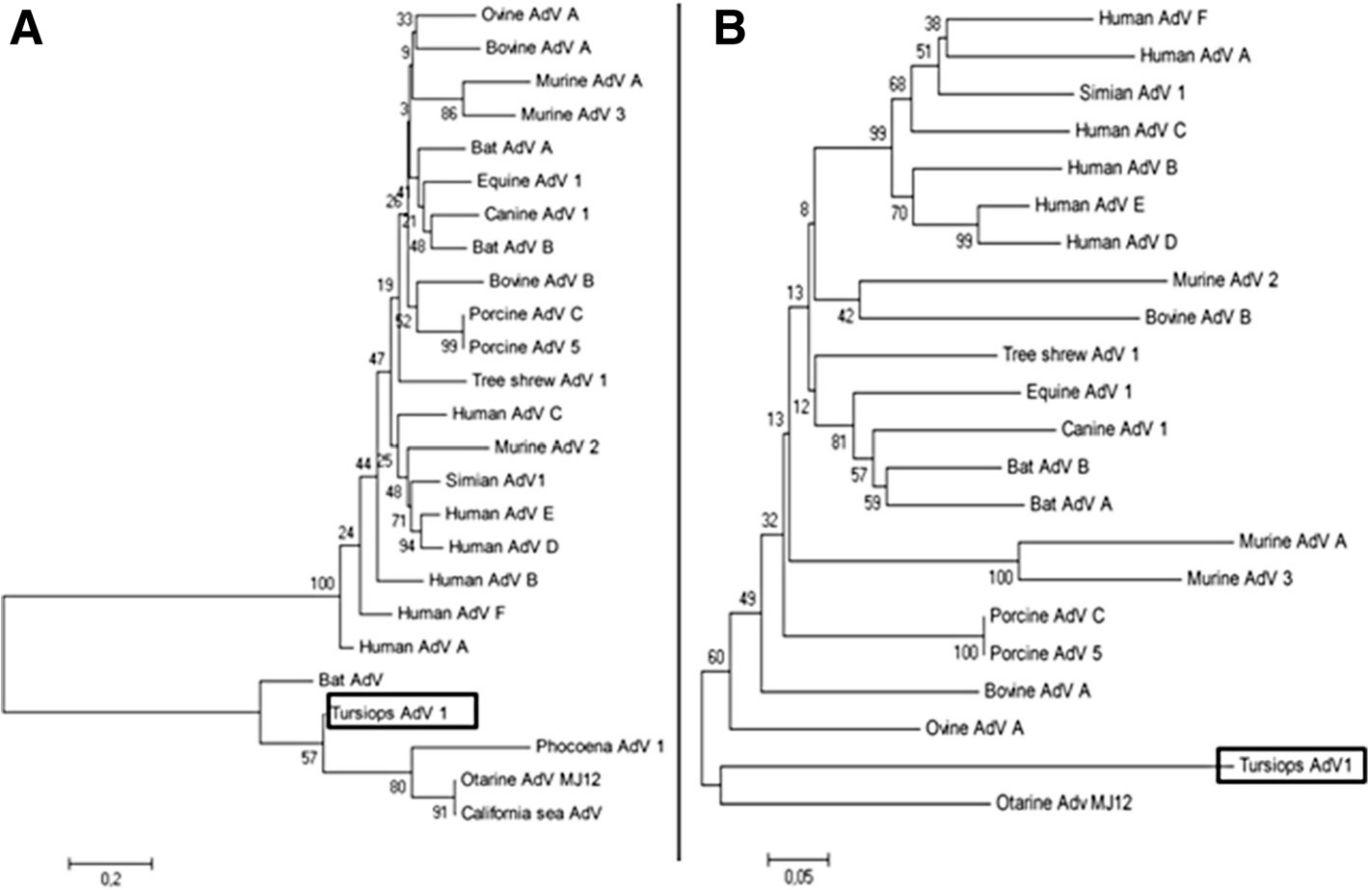
**Phylogenetic analysis of adenoviruses based on regions of genes** ***pol*** **and** ***hexon*** **of tursiops adenovirus 1.** Neighbor-joining trees were based on amino acid sequences deduced from partial sequences of genes *polymerase (pol)* **(A)** and *hexon* **(B)** from tursiops adenovirus 1 (enclosed in rectangular frames) and from other selected species of adenovirus (AdV). GenBank accession codes: Bat AdV, AB303301 (for pol); Bat AdV A, GU226970; Bat AdV B, JN252129; Bovine AdV A, NC_006324; Bovine AdV B, AF030154; California sea lion AdV 1, GU979536.1; Canine AdV 1, Y07760; Equine AdV 1, JN418926.1; Human AdV A, NC_001460.1; Human AdV B, NC_001405; Human AdV C, NC_001405; Human AdV D, AC_000006.1; Human adenovirus E, NC_003266.2; Human AdV F, NC_001454.1; Murine AdV A, NC_000942.1; Murine AdV 2, HM049560; Murine AdV 3, EU835513; Otarine AdV MJ12, AB714141 (for pol) and AB714142 (for hexon); Ovine AdV A, NC_002513; Phocoena AdV 1, JN377908.1; Porcine AdV A, NC005869; Porcine AdV 5, AF289262.1; Simian AdV 1, NC_006879; Tree shrew AdV 1, AF258784.1. The MEGA 5.2 software [29] was used to perform for the phylogenetic analysis. P-distance matrices were calculated, and tree topologies were inferred by the neighbor-joining method based on p-distances. Topology reliability was tested by bootstrapping 1000 replicates generated with a random seed. The bars at the bottom indicate relative phylogenetic distance.

The sequences of the PCR-amplified *pol* and *hexon* gene fragments (Genbank entries KJ126836 and KJ126837, respectively) were identical for all four animals, which indicates that they were all infected with the same adenovirus. Similarity searches of all adenoviral sequences in Genbank (www.ncbi.nlm.nih.gov/nuccore) using BLASTN (http://blast.ncbi.nlm.nih.gov) showed that the *pol* amplicon in the present work exhibited the highest nucleotide identity (78%) with entry JN377908, this being a deposited, but unpublished, sequence from an adenovirus detected in a harbour porpoise (*Phocoena phocoena*) on the coast of Florida (USA). The *hexon* amplicon showed the highest identity (72%) with otarine adenovirus strain MJ12 (entry AB714142) [14]. We conclude that the adenovirus in the four bottlenose dolphins closely resembles, but differed from previously detected adenoviruses in marine mammals. We designate this apparently novel adenovirus as tursiops adenovirus 1. The phylogenetic analysis [29] of the amino acid sequences deduced from the *pol* and *hexon* amplicons further supports the closeness of tursiops adenovirus 1 to the adenoviruses isolated from other marine mammals, including harbour porpoises, seals and sea lions (Figure 2).

In an effort to confirm this tursiops adenovirus 1 and characterize it in greater detail, we attempted to grow it in HeLa cells. This human-derived cell line was used as specific-host cell lines were not available, and also because canine adenovirus 2 has been proven to infect HeLa cells [30]. Centrifuged (10 min, 2,000 × g, 4°C) fecal homogenates prepared by vortexing in 3 volumes of phosphate-buffered saline (PBS), were filtered through sterile 0.2 μm-pore filters (Sartorius, Goettingen, Germany) and were left to stand for 1 h at 4°C in the presence of 0.5 mg/ml gentamycin. 0.2 ml of this solution were added to subconfluent HeLa cell monolayers, which had been grown (37°C, 5% CO_2_) in 6-well plastic plates in Dulbecco’s Modified Eagle Medium (DMEM; Lonza, Basel, Switzerland) supplemented with 10% heat-inactivated fetal bovine serum (FBS) (Invitrogen, Carlsbad, CA, USA), 100 U/ml penicillin and 0.1 mg/ml streptomycin (Sigma-Aldrich, St. Louis, USA). Immediately before this inoculation, the medium was replaced with 0.3 ml of FBS-free medium. After 1 h, 1.5 ml of the 10% FBS-containing medium was added and the culture continued. Each day, a 0.2-ml sample of culture medium was taken for the PCR *pol* gene analysis. With the cultures inoculated with samples of diseased animals, the PCR assay on the culture medium was initially negative or very weakly positive, but became strongly positive on days 4-5. The cultures inoculated with samples of healthy dolphins, which had been processed in parallel, did not give a positive PCR reaction. The sequence of the *pol* fragment amplified by PCR from the positive cultures on day five after inoculation with the fecal material was identical to that obtained directly from fecal samples, which confirmed that the virus corresponded to the original adenovirus detected in feces. These results suggest that the virus can replicate to some extent in HeLa cells. However, transmission electron microscopy (tFEI Tecnai G2 Spirit microscope, EM Service, Principe Felipe Research Centre, Valencia, Spain) of ultra-thin sections of the glutaraldehyde-fixed, osmium tetroxide-stained, durcupan-embedded and lead citrate counterstained cell monolayers on day 5 of culture did not provide conclusive evidence of adenovirus (although a few suggestive images of rounded particles of around 125-160 nm were observed [31] in the infected cells nuclei, data not shown). Therefore, the inference from these results that the virus can replicate in HeLa cells was confirmed by immunofluorescence using a monoclonal antibody against canine adenovirus 1 (clone 2E10-H2, VMRD, Pullman, WA, USA) and, as secondary antibody, Alexa 488-conjugated goat anti-mouse IgG (Invitrogen). Cells were fixed with ice-cold methanol (methanol 100%, Sigma) at -20°C and permeabilized with 0.1% Triton X-100 for 5 min. After three washes with PBS, cells were blocked with 5% goat serum in PBS for 1 hour and stained at room temperature with anti-CAV1 MoAb for 1 h at a final concentration of 0.1 mg/ml. Then, cells were washed three times with PBS and incubated for 1 h with a secondary Alexa 488-conjugated goat anti-mouse Ab (diluted 1:800) for detection in the green channel. After three washes with PBS, cover glasses were dried at room temperature for 20 minutes followed by assembly of the cover glasses in the slides. Confocal Microscopy was performed at the Madrid Science Park microscopy facility using a Olympus FV1200 equipped and images were processed with Photoshop CS5.

**Figure 3:**
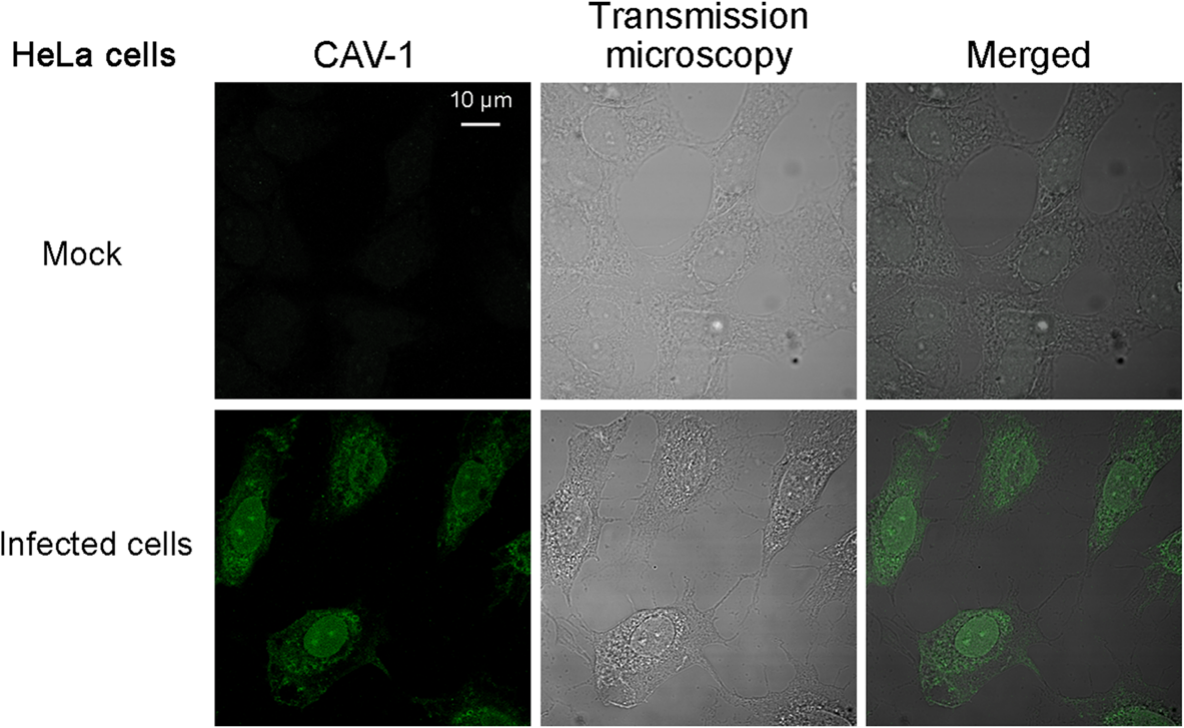
**Immunofluorescence of HeLa cell cultures inoculated with FBS medium-free (top) and fecal extracts from diseased animals (below).** The HeLa cells were fixed the day 4 after infection. Immunofluorescence staining was done using anti-Cav-1 MoAb (first column). Transmission microscopy images were used to see the location and structure of the HeLa cells (second column). The merged images shown in the last column were generated with Photoshop S5 software. Pictures were taken at 600x magnification and scale bar represents 10 um.

The cells of a culture inoculated with material derived from a sick animal (animal 3, on third day of clinical manifestations), when examined on day four after inoculation, gave clear nuclear fluorescence and moderate diffuse cytoplasmic fluorescence (Figure 3, downright panel), as previously described for adenovirus [32]. In contrast, a parallel culture inoculated with an equivalent amount of FBS- medium free material (Mock) did not exhibit substantial fluorescence (Figure 3 topright panel).

## Conclusions

In summary, we report herein self-limiting gastrointestinal disease in bottlenose dolphins, which appears to be due to a hitherto unknown adenovirus that is genetically close to the adenoviruses found previously in marine mammals, such as sea lions. Adenoviral causality is supported by the exclusive detection in the four diseased animals, concomitantly with the disease, of adenovirus in feces and of antibodies for canine adenoviruses 1 and 2 in the serum. While adenoviral infection of sea lions causes hepatitis and death [13,14], the dolphins infected with putative tursiops adenovirus 1 suffered self-limiting disease, with no signs of hepatitis. In addition, the dolphins showed no apparent respiratory or ocular pathologies, which are frequent in adenoviral infections of many other species [1,5]. Thus this adenovirus may not show liver, lung or eye tropism. Full adenoviral genome sequencing might help predict tropism, since certain genetic elements have been associated with certain tropisms [33]. The full viral sequence might also be necessary to more broadly confirm our present inference, based on the limited phylogenetic analysis of partial *pol* and *hexon* gene sequences (Figure 2), and that this adenovirus is more closely related with adenoviruses of other marine mammals than with those of other taxonomic groups. This close relation suggests that a branch of the adenoviral tree evolved when marine mammals became adenoviral hosts. It remains to be elucidated whether adenoviruses represent a serious threat to dolphins. In any case, the present findings highlight the need to consider this adenovirus a causal agent of dolphin gastroenteritis, which should be taken into account in the differential diagnosis of this condition, at least for captive dolphins.

## Abbreviations

*Pol*: *Polymerase*
HAdV: Human adenovirus
EM: Electron microscopy
CBC: Complete blood counts
qPCR: Real-time polymerase chain reaction
USA: United States of America
DMEM: Dulbecco’s Modified Eagle Medium
PBS: Phosphate-buffered saline
FBS: Fetal bovine serum
OsO4: Osmium tetroxide
